# Does *Ureaplasma* spp. cause chronic lung disease of prematurity: Ask the audience?

**DOI:** 10.1016/j.earlhumdev.2008.12.002

**Published:** 2009-05

**Authors:** Nicola C. Maxwell, Diane Nuttall, Sailesh Kotecha

**Affiliations:** Department of Child Health, Cardiff University, Cardiff CF14 4XN, United Kingdom

**Keywords:** *Ureaplasma*, Respiratory distress syndrome, Chronic lung disease of prematurity, Macrolides, Randomised clinical trial

## Abstract

*Ureaplasma* has long been implicated in the pathogenesis of both preterm labour and neonatal morbidity, particularly chronic lung disease of prematurity (CLD), but despite numerous studies, reviews and meta-analyses, its exact role remains unclear. Many papers call for a definitive randomised control trial to determine if eradication of pulmonary *Ureaplasma* decreases the rates of CLD but few address in detail the obstacles to an adequately powered clinical trial. We review the evidence for *Ureaplasma* as a causative agent in CLD, asking why a randomised control trial has not been performed. We surveyed the opinions of senior neonatologists in the UK on whether they felt that there was sufficient evidence for *Ureaplasma* either causing or not causing CLD and whether a definitive trial was needed, as well as their views on the design of such a trial. Additionally, we ascertained current practice with respect to *Ureaplasma* detection in preterm neonates in the UK. There is clear support for an adequately powered randomised controlled clinical trial by senior neonatologists in the UK. There are no reasons why a definitive trial cannot be conducted especially as the appropriate samples, and methods to culture or identify the organism by PCR are already available.

## 1 Introduction

The role of *Ureaplasma* in neonatal morbidity and mortality has long been controversial and its role as a neonatal pathogen has been studied and questioned since the late 1970s [1]. *Ureaplasma* spp. has been implicated in the pathogenesis of both preterm labour and neonatal morbidity, particularly chronic lung disease of prematurity (CLD), but its exact role remains unclear. We review the evidence especially asking why an adequately powered randomised control trial to determine the causative role of *Ureaplasma* in the pathogenesis of CLD has not been performed.

## 2 *Ureaplasma* spp.

*Ureaplasmas* are eubacteria which belong to the class Mollicutes. They do not have cell walls and are thought to be the smallest free-living, self-replicating cells. They are limited by the lack of cell wall to a parasitic existence in eukaryotic cells. *Ureaplasmas* were previously designated “T-mycoplasma” but later the genus *Ureaplasma* was designated in view of their use of urea as a metabolic substrate. *Ureaplasma urealyticum* was the only species known to infect humans and this species was recently subdivided into 2 separate species, *U. urealyticum* and *U. parvum*, on the basis of their 16S ribosomal RNA gene sequences.

## 3 *Ureaplasma* in the antenatal period

Up to 80% of women have been reported to be colonised with genital mycoplasmas [2]. *Ureaplasma* spp. has been shown to be implicated in preterm labour, spontaneous abortion and still birth [1]. It has also been recorded as the most common organism isolated from the amniotic fluid of women in preterm labour, even with intact membranes [3,4]. As many as 22% of women with PROM or preterm labour have been shown to have evidence of *U. urealyticum* in their amniotic fluid [5].

Transmission of *Ureaplasma* from a colonised or infected mother may depend on a number of variables: particularly gestation, birthweight, route of delivery and preterm prelabour rupture of the membranes. Preterm infants appear to be more likely to become colonised than their term counterparts [6] with the rate of vertical transmission ranging between 18% and 55% for term infants and 29% and 55% for preterm infants [7].

There may be a higher risk of vertical transmission of *Ureaplasma* in babies of lower birth weight, for example in babies weighing < 1000 g at birth transmission may be up to 89% [8] but a transmission rate of only 15% has been reported among infants of > 1500 g birthweight [9]. Alfa et al. showed that very low birth weight (VLBW, < 1500 g) infants were at significantly higher risk of acquiring *Ureaplasma* spp. in their respiratory tract than larger preterm infants [6]. The underlying cause of preterm delivery and mode of delivery have been shown to be associated with *Ureaplasma* transmission to the newborn. Aaltonen et al. [10] reported a series of 49 infants born at < 30 weeks gestation, in which 45% of 33 spontaneous deliveries were colonised with *Ureaplasma* but none of the electively delivered infants appeared to be colonised. Goldenberg et al. [11] also reported a higher prevalence of *U. urealyticum* or *Mycoplasma hominis* in umbilical cord blood among 351 mother/baby pairs at 23–32 weeks where infants had delivered spontaneously rather than electively. Infants with *Ureaplasma* are more likely to have been born following preterm prelabour rupture of membranes than those not colonised [12].

## 4 *Ureaplasma* in the newborn infant

*Ureaplasma* has been implicated in neonatal morbidity and mortality including congenital pneumonia, preterm delivery, low birth weight and intrauterine growth retardation [1] and it is thought to infect or colonise up to 37% of newborns [13]. It has been implicated in the pathogenesis of neonatal pneumonia and meningitis and there are also reports of a systemic inflammatory response in babies with evidence of *Ureaplasma* in the lower respiratory tract as evidenced by an elevated white blood cell count, particularly in the first 2 days of life [14,15].

There have been a number of case reports of *Ureaplasma* infection of the cerebrospinal fluid (CSF). Waites et al. [16] suggest that *Ureaplasma* spp. may in fact be a relatively common cause of CSF infection in the neonatal population. Their study, along with other case reports, records a range of central nervous system clinical outcomes for infants in whom such an infection is diagnosed — from spontaneous resolution [17], to development of hydrocephalus or intra-ventricular haemorrhage [18], to mortality in association with erythromycin resistant *Ureaplasma* spp. [19]. A single case report in 2002 [20] has also implicated *Ureaplasma* in the formation of a brain abscess in a neonate.

Cultrera et al. [21] studied the relationship between neonatal respiratory distress syndrome (RDS) and *Ureaplasma* in 50 babies of < 37 weeks gestation. Fifteen out of 24 babies with RDS had *U. urealyticum* or *U. parvum* detected and only 4 of 26 babies without RDS were colonised with either organism, thus suggesting that *Ureaplasma* plays a role in the development of RDS. In contrast, Hannaford et al. [22] showed significantly decreased incidence of RDS in infants of < 28 weeks gestation who were colonised with *Ureaplasma* but many of the colonised infants progressed to develop CLD which is in keeping with data from Watterberg's group showing an association between chorioamnionitis and development of CLD [23].

## 5 Chronic lung disease of prematurity

The association between the presence of *Ureaplasma* and the development of CLD remains controversial and hotly debated. The pathogenesis of CLD is multi-factorial with prematurity, ventilator induced lung injury, oxygen therapy, patent ductus arteriosus, fluid balance and infection, both ante- and post-natal, all appearing to have a role to play. It is often difficult to dissect out the effect of one particular risk factor as there is overlap between the risk factors in this multi-factorial disease. Pulmonary *Ureaplasma* colonisation is strongly linked to preterm delivery and the question remains if this pulmonary colonisation with *Ureaplasma* is an independent risk factor for CLD.

There have been numerous studies investigating the role of *Ureaplasma* in the development of CLD, as well as many reviews and meta-analyses published in recent years. The consistent observation in many publications is the difficulty in interpreting evidence from available studies due to small sample sizes, vastly different inclusion criteria, different methods of sampling and testing, different diagnostic criteria for various outcomes including CLD. Many call for definitive studies but few address in detail the obstacles to an adequately powered clinical trial.

In 1995, a meta-analysis by Wang et al. included 1479 babies from 17 studies [24] reporting a significant association between CLD diagnosed at 28 days of life and *Ureaplasma* colonisation with an overall relative risk of 1.72 (95% confidence intervals 1.5–1.96). Many studies at the time did not use a definition of oxygen dependency at 36 weeks to diagnose CLD thus association with this outcome was not available.

Since that review, several further studies have been completed, including one by Kotecha et al. [25] who sought *Ureaplasma* in bronchoalveolar lavage fluid from 17 preterm neonates without clinical or laboratory evidence of infection in either the mother or infant and reported that 6 were positive for *U. urealyticum*. Of the 6 babies with *Ureaplasma*, 5 developed CLD whereas only 4 of the 11 babies without *Ureaplasma* developed CLD. Their data strongly implicated *Ureaplasma* in the development of the pulmonary inflammatory response observed in infants who progress to develop CLD. In a cohort of 126 preterm deliveries, Kafetzis et al. [9] found a significant increase in CLD as well as mortality among *Ureaplasma* colonised infants. van Waarde et al. [26] found that *Ureaplasma* was significantly associated with both CLD and lower gestational age but logistic regression analysis failed to show a correlation between *Ureaplasma* colonisation and CLD. They highlight the need for correction for gestation in this sort of study and note several studies which had found significant relationships between *Ureaplasma* and CLD where this adjustment had not been made.

In 2005, a further meta-analysis by Schelonka et al. [27] including 23 studies and 2216 babies showed an odds ratio of 2.83 (95% confidence intervals 2.29–3.51) for the relationship between the presence of *Ureaplasma* and CLD diagnosed at 28 days of life. There were 751 babies for whom data was available regarding CLD at 36 weeks gestation, again this showed a significant association. However, they noted that studies with small numbers were more likely to influence the association suggesting publication bias against studies which perhaps did not show an association.

More recent studies continue to fuel the controversy: Pandey et al. [12] reported no role for *Ureaplasma* in the development of CLD in a group of 100 babies of < 34 weeks gestation. Goldenberg et al. [11] studied 351 mother/baby pairs at 23–32 weeks, where the umbilical cord blood showed evidence of *Ureaplasma* or *M. hominis* and a probable association of *U. urealyticum* with the development of CLD. In other studies, *U. urealyticum* colonised infants have shown a non-significant trend towards higher neonatal morbidity (longer ventilation, longer hospital stay, younger gestational age, higher incidence of CLD and more late onset sepsis) [5].

The role of *Ureaplasma* in CLD is further complicated by the identification of different patterns of *Ureaplasma* colonisation in the preterm neonate (persistently positive, early transient, late acquisition) which may also impact on the likelihood of a colonised neonate developing CLD, with only the “persistently positive” group showing a higher rate of CLD [28].

## 6 Obstacles to a clinical trial

The lack of an optimal sample and method of identification of *Ureaplasma* have perhaps contributed the most to the lack of an adequately powered clinical trial to determine if eradication of *Ureaplasma* decreases the rates of CLD. The optimal clinical sample for detection of *Ureaplasma* infection or colonisation remains unclear. Clinical samples reported in the literature vary from amniotic fluid [5,29] taken at antenatal amniocentesis through to cord blood [11] and placental swabs or tissue [30–32] taken at delivery to gastric aspirates at delivery or later endotracheal, bronchoalveolar and nasopharyngeal samples [25,31] or even neonatal blood samples [21]. Some of the contradictory findings in the literature may be as a result of different sampling methods and different diagnostic tests being applied. Most satisfactory samples are likely to be either tracheal fluid or gastric fluid [33].

Similarly the best test to identify *Ureaplasma* is not entirely clear. Traditional culturing techniques have been cumbersome but modern techniques such as the polymerase chain reaction (with quantitation by real time PCR) show great promise [34] especially after an initial period of culture for 24–48 h. Our preliminary data has shown that culture is required only for 24–48 h to identify the presence of *Ureaplasma* in most infants. Subsequent use of PCR is mainly for epidemiological and research purposes including identification of the serovar and mechanisms of antibiotic resistance.

## 7 Treatment of *Ureaplasma* for prevention of CLD

If *Ureaplasma* has a causative role in the pathogenesis of CLD, it would be reasonable to expect the incidence of CLD to decrease by eradicating its colonisation with antibiotic treatment. Erythromycin is the most commonly used antibiotic for eradication of *Ureaplasma* [35], although contradictory results of such treatment have been reported. A 5–10 day course of treatment failed to eradicate *Ureaplasma* in 55% of very low birthweight infants studied by Baier et al. [36] however, an earlier study found almost all patients became culture negative after a 7–10 day course [37]. An additional important point is the lack of information on antibiotic resistance of *Ureaplasma* to conventional antibiotics used in neonatal practice.

Once again the literature contains a number of small sample size studies which vary in the time of commencement of treatment, type and duration of antibiotic therapy and many lack documentation of eradication of the organism at the end of the course of treatment. However, only two randomised controlled trials have been included in the Cochrane review by Mabanta et al. [38] examining studies investigating the treatment of *Ureaplasma* to decrease the rate of CLD. In the first study, Lyon et al. [39] treated infants prior to knowing their colonisation status and showed no change in the number of infants who developed CLD. In the second study, Jonsson et al. [40] treated those infants with positive cultures from endotracheal or nasopharyngeal samples and were able to show a reduction in colonisation but not CLD. Together these two studies included only 37 colonised patients (clearly poorly powered to address the underlying question of whether treatment of *Ureaplasma* can decrease the rates of CLD) and there was no significant reduction in CLD with treatment in either study — disparate study designs prevented the results from being combined in the meta-analysis. Neither study reported any adverse effects of a 7–10 day course of erythromycin.

Macrolides are of great interest, not only because of their anti-microbial activities against Mollicutes such as *Ureaplasma*, but also because of their anti-inflammatory actions acting via a variety of pathways [41]. Pharmacokinetics of erythromycin in preterm infants has previously been published [37] and the mean inhibitory concentration (MIC) for clinical isolates of *Ureaplasma* from isolated from newborn infants has also been reported [42]. Interestingly Ballard and Shook have also conducted a pilot study to assess the feasibility of azithromycin as an anti-inflammatory [43].

## 8 Neonatologists' opinion on the role of *Ureaplasma* in the development of CLD

Despite many papers studying the role of *Ureaplasma* in the pathogenesis of CLD, it is perhaps surprising that an adequately powered randomised control trial has not been performed [38]. Reasons behind the lack of such a trial may include technical ones (e.g. the best sample, culture techniques) or individual views of clinicians perhaps believing that *Ureaplasma* does not cause respiratory disease in preterm newborn infants. We therefore sought the views of senior clinicians in the United Kingdom (UK) caring for newborn infants in dedicated neonatal units. In particular, we were interested if they thought, from their own reading, that there was sufficient evidence of *Ureaplasma* causing or not causing CLD, and if they had views on the necessity of a definitive but sufficiently powered randomised control trial to determine if eradication of *Ureaplasma* decreases the rates of CLD.

## 9 Survey method

We designed a structured questionnaire consisting of 18 questions each with a choice of possible answers and mailed it to 300 UK consultant neonatologists or paediatricians with a special interest in neonatology. A second reminder was sent to those who did not reply within 4–6 weeks.

Questions covered four main areas:

• Their opinion of the current evidence for *Ureaplasma* causing or not causing CLD
• Their own current practice for detecting *Ureaplasma*
• If in their opinion a definitive trial to determine “if *Ureaplasma* spp. causes CLD” was necessary?
• Their views on the design of a trial.

## 10 Survey results

Of the 300 questionnaires sent out, 172 (57%) were returned. Of the 172 respondents, 137 were consultant neonatologists while the remainder were paediatricians with a special interest in neonatal medicine. One hundred and twenty five stated that they worked in level 3 intensive care unit and only 7 worked in level 1 units. Questionnaires were sent to clinicians in 97 different neonatal units. Of these, a response was received from at least one consultant in 82 units. The units from which no responses were received included six level 1 units, seven level 2 units and two level 3 units. Thus the majority of neonatal units doing intensive care responded and most level 1 and 2 units also responded from the majority of units. When analysed separately there were no particular differences of opinion between the staff from units providing differing level of intensive care.

**Fig. 1:**
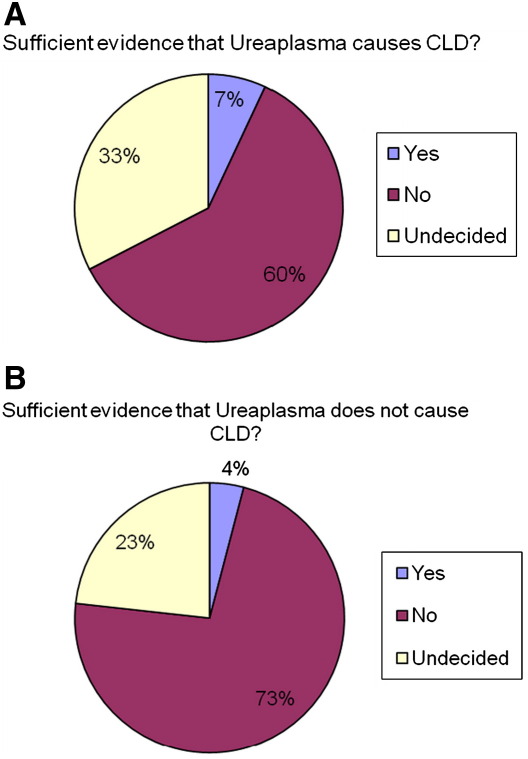
Clinicians' opinion of whether *Ureaplasma* causes CLD or not.

When asked if they thought there was enough evidence showing if *Ureaplasma* caused CLD, of those who gave an opinion, 12 (10% of those with an opinion) thought there was but the majority did not (104, 90%) with the remainder being undecided (Fig. 1A). Interestingly, 125 (of 132 with an opinion, 95%) did not think there was sufficient evidence that *Ureaplasma* did not cause CLD with only 7 (5%) thinking there was (Fig. 1B).

**Fig. 2:**
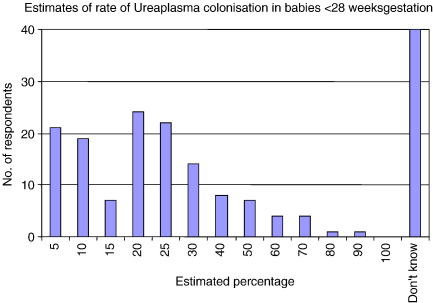
Clinician estimates of colonisation with *Ureaplasma* in infants born at less than 28 weeks gestation.

**Fig. 3:**
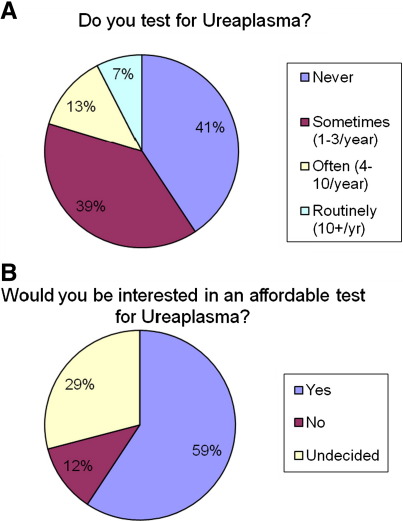
Whether clinicians routinely tested or would be interested in an affordable test for identifying *Ureaplasma*.

When asked to estimate the rate of *Ureaplasma* colonisation in infants of < 28 weeks gestation, most neonatologists estimated between 5 and 25% (range 0–90%, mode 20%) (Fig. 2). This estimate was unlikely to have been based on personal clinical experience as only 21% of neonatal units reported testing for *Ureaplasma* either often or routinely (> 3 samples/year) (Fig. 3A). Although our questionnaire did not specifically explore the reasons for the low rate of testing for *Ureaplasma*, many respondents added free text stating that testing for *Ureaplasma* was either too expensive or that the culture results were often delayed thus not being useful in clinical practice. It was therefore interesting to note that 102 of 122 (84%) who gave a response (with 50 undecided) would be interested in an affordable test to identify the organism (Fig. 3B).

**Fig. 4:**
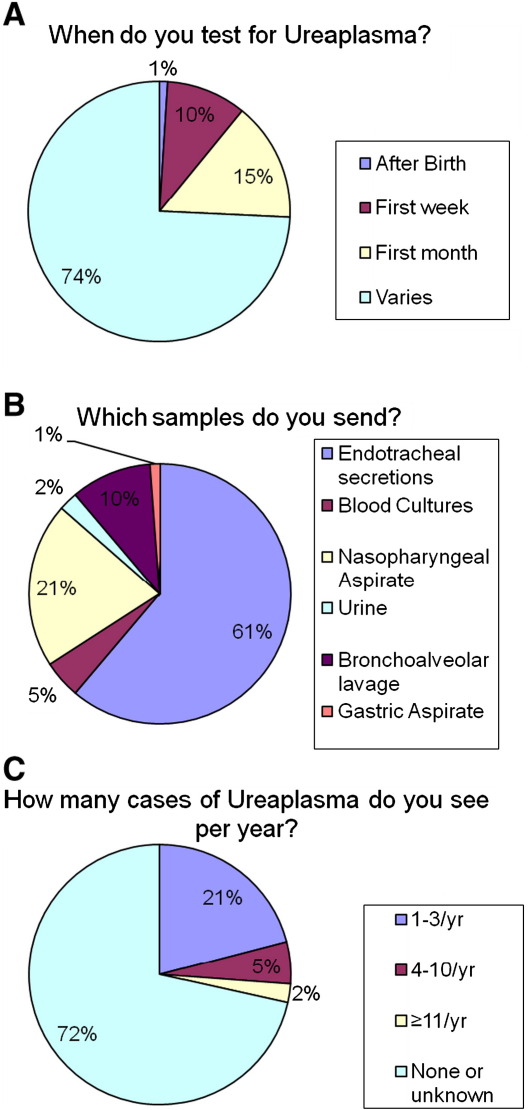
Frequency of local testing and nature of sample sent for identifying *Ureaplasma* as well as how often *Ureaplasma* is seen in their own units.

Of the 102 respondents who tested for *Ureaplasma*, the sample type varied widely as did the timing of the sampling. As the evidence for the role of *Ureaplasma* in the development of CLD remains uncertain, it is perhaps not surprising that samples were sent for testing according to the clinical status of the infant rather than an expectation of the sample being positive (Fig. 4A). Unsurprisingly, endotracheal secretions were the most frequent sample sent for testing but others including blood culture were also sent by some units (Fig. 4B). Many units sent more than one sample type.

Only a few respondents were able to estimate the number of infections seen by their own unit (28%) with most saying they were unable to answer the question due to lack of routine testing (Fig. 4C). Many respondents stated that they did not test for *Ureaplasma* because a specific test was not available locally from their local microbiology service and that results were not readily available if sent to regional or supra-regional laboratories. They clearly suggested that the results should be available within a reasonable time and should be affordable.

Having ascertained current practice, we proceeded to seek opinion of whether a definitive randomised trial to formally assess the role of *Ureaplasma* in the development of CLD was thought necessary. From the 135 who gave an opinion, 117 (87%) thought that a clinical trial was necessary but 37 did not register an opinion. From 79 who had an opinion (with 38 undecided), 76 (96%) stated that they would be interested in participating in a future clinical trial.

**Fig. 5:**
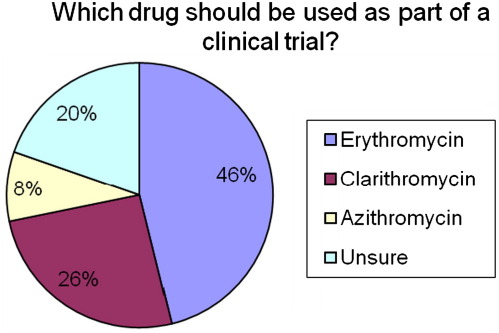
Clinicians' opinion of what drug should be used in a clinical trial to test if eradication of *Ureaplasma* decreases the rates of CLD.

The majority (90/117) of respondents who were in favour of a clinical trial suggested that infants between 23 and 28 weeks gestation should be included in a trial and that erythromycin was their drug of choice within a trial (Fig. 5). Currently in the UK erythromycin is readily available for neonatal use in both intravenous (IV) and oral formulations whereas azithromycin is only available as an oral preparation (although available as an intravenous preparation in the US) and experience with clarithromycin in the newborn is limited.

As far as the timing of commencing antibiotic treatment was concerned, there was an equal divide between those who wanted to wait for culture results before starting treatment and those who thought that treatment should start immediately at birth. Many suggested starting early as the inflammatory process would have been firmly established by the time culture results were available.

The duration of treatment favoured by UK neonatologists was 7–14 days (range 3–28 days, mode 14 days) and when asked if they had any concerns with antibiotic treatment, 51 were concerned, 55 were not and 11 were unsure. Concerns, when registered, were mainly about gastrointestinal effects particularly with erythromycin; antibiotic resistance; alterations to normal microbial flora or phlebitis in the smallest preterm infants.

## 11 Conclusions

Despite many studies investigating the role of *Ureaplasma* in the development of CLD, controversy continues partly due to the lack of an adequate clinical trial to confirm or refute the association. Repeated meta-analyses implicate the association between *Ureaplasma* and CLD but the Cochrane review [38] could only include two very disparate studies with very small numbers stating that even a large effect would have been missed by the trials included. There is clear support for a clinical trial at least by senior neonatologists in the UK. There are no reasons why a definitive trial cannot be conducted especially as the appropriate samples (endotracheal or gastric fluid) and methods to culture or identify the organism by PCR are already available.
